# The impact of team based interprofessional comprehensive assessments on the diagnosis and management of diabetic foot ulcers: A retrospective cohort study

**DOI:** 10.1371/journal.pone.0185251

**Published:** 2017-09-26

**Authors:** Ranjani Somayaji, James A. Elliott, Reneeka Persaud, Morgan Lim, Laurie Goodman, R. Gary Sibbald

**Affiliations:** 1 Department of Medicine, University of Calgary, Calgary, Canada; 2 Toronto Regional Wound Healing Clinic, Mississauga, Canada; 3 Trillium Health Partners, Institute for Better Health, Mississauga, Canada; 4 Institute of Health Policy, Management and Evaluation, University of Toronto, Toronto, Canada; 5 Humber River Hospital, Toronto, Canada; 6 Trillium Health Partners, Mississauga, Canada; 7 Dalla Lana Faculty of Public Health, University of Toronto, Toronto, Canada; 8 Women’s College Hospital, Wound Healing Clinic, Division of Dermatology, Department of Medicine, Toronto, Canada; Weill Cornell Medical College in Qatar, QATAR

## Abstract

**Background:**

Diabetic foot ulcers (DFU) are increasingly prevalent, and associated with significant morbidity, mortality, and cost. An interprofessional approach to DFU management is critical given the etiological complexity involved. This study aimed to assess the impact of an interprofessional team approach on DFU diagnosis and management for a cohort of patients receiving treatment in an Ontario Canada home care setting.

**Methods:**

A retrospective cohort study of patients attending a large regional Community Care Access Centre (CCAC) between February 11, 2013-September 30, 2014 was conducted. Following CCAC referral, patients were assessed by an interprofessional team at the Toronto Regional Wound Healing Centre (TRWHC). Those aged > 18 years with a DFU of > 6 weeks duration were included. The primary outcome was the precision of the initial diagnosis relating to DFU etiology (i.e. neuropathic, ischemic or mixed etiology). Secondary outcomes included wound healing, and infection parameters. Analysis was completed with STATA 13.1 (College Stn., TX) of pre-determined outcomes with 2 sided α of 0.05.

**Results:**

A total of 308 patients were screened, and 49 patients (67.3% male) of mean age 64.2 years (SD 13.7) with a diagnosis of DFU > 6 weeks duration were included for analysis. Of these, 95% were referred with unspecified DFU, and were reclassified to a precise diagnosis relating to etiology, including neuropathy, ischemia or neuroischemic etiology following TRWHC assessment (p < 0.001). For secondary outcomes post-assessment, healability assessment was conducted for a greater proportion of patients (100% versus 44%, p < 0.001). Infection was identified in a greater number of patients (p = 0.04), and of the 35 patients, 94.5% had deep and surrounding infection, and 88.0% were initiated on systemic antibiotics. Vascular insufficiency was diagnosed in an additional 14.3% of the cohort (p = 0.03). Offloading/footwear assessment was conducted in all patients compared with 30.6% prior to referral (p < 0.001) Dressing change frequency decreased significantly following TRWHC assessment (pre: 4.31/week; post: 3.54/week; p = 0.03). Pain scores decreased (2.18 to 1.67) on the numerical rating scale but this was not statistically significant at the final TRWHC assessment. Notably, 36.7% (18/49) reported improved quality of life by the second TRWHC encounter.

**Conclusions:**

Interprofessional care teams are associated with improved diagnostic acumen and wound healing outcomes over conventional community care services. Initiatives including best practice interprofessional diabetic foot care pathways are recommended with timely vascular management of ischemia, treatment of deep and surrounding infection as well as the availability of foot care and footwear.

## Introduction

Diabetes mellitus is a non-communicable chronic disease impacting approximately 415 million individuals worldwide [1]. Global diabetes prevalence continues to rise and in 2014 it was estimated 9% of all adults had the disease [1,2]. Further, it is estimated that 50% of persons with diabetes are unaware of their diagnosis, and that diabetes is directly attributed to more than 1.5 million deaths annually [3]. Individuals with diabetes have higher rates of health care utilization, hospitalization, and have a two-fold risk of death at each age group [4–6]. Canada has > 2.4 million individuals living with diabetes and the province of Ontario has one of the highest age-standardized prevalences in the country [7].

Non-traumatic lower extremity amputations are a common complication of diabetes. Approximately 85% of all amputations are associated with preceding diabetic ulcers of the skin [8,9]. Diabetic foot ulcers (DFU) occur in up to 25% of people with diabetes and it is estimated that a lower extremity is amputated every 20 seconds due to diabetes [10–12]. DFUs are commonly of a neuropathic, vascular, or a combined (neuro-ischemic) etiology, and are often chronic in nature [13]. Furthermore, DFUs and resultant amputations are associated with significant morbidity, mortality, emotional damage, and financial costs [11, 14, 15]. Therefore an organized approach to DFU screening, prevention, and management is critical for cost savings to healthcare systems [16].

It is well established that glycemic control, and preventative foot care in high risk patients are effective diabetes management interventions [2, 16]. However, once an individual develops a DFU, the optimal approach and team structure is less well established. DFU prevalence is estimated at 90, 000 in Ontario resulting in annual costs upwards of 500 million dollars (CAD) for community care alone [17,18]. As 10 to 15% of persons with diabetes will develop a DFU in their lifetime, the DFU prevalence and associated costs will only continue to increase. Growing evidence demonstrates interprofessional teams defined as a group of individuals from different health care discriplines that function in a collaborative and integrated structure to be the optimal approach for care of complex chronic wounds, including DFUs, and have also demonstrated reductions in incidence, and subsequent complications including amputations [19–23]. Further, international initiatives such as the Guyana Diabetes and Foot Care program were able to demonstrate that an interprofessional approach utilizing assessment of vascular status, presence of infection, and plantar pressure redistribution (‘V.I.P’) reduced lower limb amputations by up to 80% [24].

Ontario’s community based wound care services are administered through a publicly funded single-payer home care system coordinated by Community Care Access Centres (CCAC). The CCAC system is structured into 14 geographically discrete areas, and primarily delivers care via contracts with service provider organizations (SPO) in patient homes and outpatient clinics. The care is largely nursing based and is provided on the basis of physician orders from hospital and outpatient care settings. There have been reports of inconsistent specialist wound team consultations or follow-up [25], leading to lack of standardization, and coordination of wound care within the system. For DFU management, strategies that have been studied and validated include the ‘wound bed preparation’ paradigm which utilizes the ‘V.I.P.’ assessment approach, and includes vascular, infectious, and pressure redistribution components for determination of etiology, and treatment parameters [26]. Thus, we aimed to assess the diagnostic and management impact of an interprofessional team approach on DFU care in Mississauga Halton, a health region in Ontario, Canada.

## Methods

### Setting and population

In Ontario, Mississauga Halton is a large geographic area where community care services are primarily served currently by a CCAC clinic in the home or outpatient clinic settings. The Toronto Regional Wound Healing Centre (TRWHC) is an interprofessional outpatient clinic located in Mississauga Ontario that provides comprehensive assessment and care plan services for patients with complex wounds. The interprofessional team consists of a physician with training and extensive experience treating complex wounds, three nurses with advanced wound care expertise, a chiropodist trained in providing appropriate offloading, and a certified diabetes educator. Initial comprehensive assessments are approximately two hours long, and utilize both interview and physical assessment methods as outlined in the Wound Bed Preparation paradigm [26]. Patients were assessed by the nurse, physician, and diabetes educator as a minimum, and by the chiropodist if offloading was required (on the basis of wound etiology and location). Patients are assessed within ten business days following a referral. Follow-up appointments are scheduled as necessary and guidance is provided for ongoing wound care. Written consent on standard forms was obtained from participants, and the study protocol was approved by the Trillium Health Partners Research Ethics Board (REB-ID635).

### Patient selection and study design

A retrospective cohort study of patients referred to the TRWHC from a local CCAC between February 11, 2013 and September 30, 2014 was conducted. Patients who were aged ≥ 18 years with history of diabetes mellitus, and a referral diagnosis of DFU, and who were seen at least once at the TRWHC were included in the analysis. The diagnosis of diabetes mellitus was confirmed through review of chart records of glycemic testing, including hemoglobin A1c (HbA1c) values and/or previously recorded diagnosis. As patients may have multiple ulcers, the primary ulcer was designated by the CCAC staff of chief concern based on standardized wound parameters including size, depth, location, and complications (i.e. infection, vascular compromise). Demographic and clinical data were collected through chart review of CCAC referral forms, laboratory investigations, and TRWHC medical records using a case report form. The case report form was tested using ten randomly selected patient charts and reviewed for changes by three investigators separately prior to finalizing the form (JE, RP, RGS). Collected data included: demographics (age, sex), co-morbidities, smoking status, referral details, vascular status (including Doppler testing), presence of infection, wound parameters (duration, size, location, treatment to date, healability classification), diabetes history and complications, footwear or offloading details, functional ability, and TRWHC diagnosis and treatment ordered. When discrepant information was noted between CCAC and TRWHC records, the TRWHC chart data was utilized.

### Statistical analysis

Continuous variables were described using means and standard deviations (SD), and discrete or non-normally distributed variables were described with medians and interquartile ranges (IQR). Comparisons were conducted with Student t-tests and Chi-squared tests for continuous and categorical variables respectively. For the primary outcome of the precision of DFU diagnosis, Chi-squared testing, and inter-rater reliability with Cohen’s kappa was used to compare the CCAC and TRWHC diagnoses. Precision of DFU diagnosis was coded as a binary variable at the time of TRWHC assessment. A precise diagnosis was defined as one which included etiology in the terminology, such as neuropathic, infectious, or vascular aspects relating to an otherwise unspecified ulcer, or unspecified DFU diagnosis. Chi-squared analysis was utilized to analyse the secondary outcomes of assessment of healability (defined as healable, maintenance, non-healable), vascular compromise (yes/no), identification of infection (deep and surrounding or superficial infection; bacterial damage present yes/no), assessment of pain (yes/no), footwear/offloading assessment (yes/no + qualitative assessment of devices) and wound closure events (yes/no). Infection was defined as critical colonization if superficial, or deep and surrounding wound infection based on previously validated NERDS and STONEES criteria [26]. Student’s t-test with unequal variance was utilized to analyse mean weekly dressing change frequency. Inter-rater reliability with Cohen’s kappa was also used to compare healability classification. All outcomes were determined prior to data collection and hypotheses were two sided with an alpha significance of 0.05. Analyses were conducted with STATA 13.1 (College Stn., Texas).

## Results

A total of 318 patients referred to the TRWHC were screened, and 49 patients (67.3% male) with diabetes mellitus referred with DFU were included for analysis (*Table 1*). Baseline demographics of age (p = 0.09) and sex (p = 0.08) were not significantly different from the screening cohort. The patients had an overall mean age of 64.2 years (SD 13.7), with a median wound duration of 26.0 weeks (IQR 10–52), and a median of 3.5 (IQR 3–7) clinical encounters per week through the CCAC at the time of TRWHC referral. Referrals to the TRWHC were most commonly initiated by Enterostomal therapists/wound care specialists (28/49, 57.1%), and were requested on a priority or urgent basis defined as < 7 days duration (30/49, 61.2%); all patients were seen within ten business days following referral. Following TRWHC assessment, patients were followed for a median of 12.0 weeks (IQR 5–25) and attended a median of 3.0 nursing homecare treatment visits (IQR 2–5) per week. Of the cohort, only 18% (7) had a BMI value in the normal adult range (18.5–24.9), with the majority being obese. Most common other co-morbidities were hypertension (67.4%), dyslipidemia (55.1%), current or historical tobacco use (38.8%), peripheral vascular disease (34.7%), and heart disease (24.5%). Approximately 30% of the cohort had a history of prior DFU associated surgical interventions for either the presenting ulcer, or a past ulcer. Notably, less than one-third of the cohort had received adequate foot screening, foot care (including improper footwear devices without modifications), or diabetes related testing (i.e. HbA1c) at the time of referral. Additionally, less than 5% of patients had vascular assessment or need for vascular testing documented when they were referred.

**Table 1:** Baseline characteristics of study cohort at initial comprehensive assessment.

Parameters		Cohort n = 49
Wound duration (weeks), median (IQR)		26.0 (10–52)
Wound size (cm^2^), median		1.8 (0.6–7.0)
Male sex, No. (%)		33 (67.3%)
Age, mean (SD), y		64.2 (13.7)
Body Mass Index*, median (IQR)		28.7 (25.8–32.0)
Diabetes mellitus, No. (%)		49 (100.0%)
DFU complications, No. (%)		
	DFU surgical interventions	15 (30.6%)
	History of foot amputation (digit +/- forefoot)	8 (16.3%)
Comorbidities, No. (%)			
	Current or historical smokers	19 (38.8%)
	Heart disease	12 (24.5%)
	Peripheral vascular disease	17 (34.7%)
	Renal insufficiency	16 (32.7%)
	Hypertension	33 (67.4%)
	Dyslipidemia	27 (55.1%)
	Known malignancy	4 (8.2%)
	Arthritis	3 (6.1%)
Completed components at time of CCAC referral, No. (%)			
	Recent HbA1c measurement	5 (10.2%)
	Neuropathy testing	0 (0.0%)
	Footwear assessment	11 (22.4%)
	Recent foot specialist assessment	11 (22.4%)
	Provision of adequate foot care	5 (10.2%)
	Provision of offloading footwear device	15 (30.6%)

Legend: SD–standard deviation; IQR–interquartile range; DFU–diabetic foot ulcer; CCAC–community care access centre

* BMI is calculated as weight in kilograms divided by height in meters squared.

### Primary outcome

Of 49 patients, only 3 patients had a precise DFU diagnosis relating to etiology at the time of referral compared with 42 patients following initial TRWHC assessment (p < 0.001). Cohen’s kappa to assess inter-rater agreement using a two unique rater model was poor with a kappa statistic of 0.02 (p = 0.24). The most common CCAC referral diagnosis was “*unspecified diabetic foot ulcer*” (77.6%). Following the TRWHC assessment, the diagnoses were further refined into ischemic, neuropathic, and mixed DFU etiologies.

### Secondary outcomes

Of the cohort, *healability* was assessed at the CCAC in 44.9% of patients, and increased to 100.0% by the first TRWHC assessment (p < 0.001) (*Table 2*). The inter-rater reliability of healability assessment between the CCAC and TRWHC was poor, with a kappa-value of 0.06. As all patients seen at the TRWHC underwent vascular assessment (Ankle-Brachial Index and/or Doppler studies), a greater proportion were identified to have vascular compromise as compared with the time of referral (p = 0.03). Similarly, assessment for bacterial damage/infection (p = 0.04), and wound associated pain (p < 0.001) was completed for a greater proportion following TRWHC assessment. Of the 35 patients identified with bacterial damage, 94.5% (n = 33) were diagnosed with deep and surrounding infection, and 88.0% were initiated on systemic antibiotics. Offloading assessments were conducted for all patients at the TRWHC as part of their comprehensive evaluation (p < 0.001). No validated pain scale score was documented at time of CCAC referral. Mean pain score on a validated visual analog scale (0–10) at time of TRWHC assessment was 2.18 (SD 3.21). Mean pain scores decreased over the follow-up period at the TRWHC, though the reduction was not significant.

**Table 2:** Wound care diagnostic and management outcomes by site.

Outcome	CCAC (Home care)	TRWHC (Interprofessional team)	p-value
Precise diagnosis, No. (%)	3 (6.12%)	42 (85.71%)	**p < 0.001**
Healability classification complete, No. (%)	22 (44.90%)	49 (100.0%)	**p < 0.001**
Vascular compromise identified, No. (%)	1 (2.04%)	7 (14.28%)	**p = 0.03**
Bacterial damage identified, No. (%)	21 (42.86%)	35 (71.4%)	**p = 0.04**
Pain assessment complete, No. (%)	4 (8.16%)	49 (100.0%)	**p < 0.001**
Footwear/Offloading assessment, No. (%)	15 (30.60%)	49 (100.0%)	**p < 0.001**
Wound closure, No. (%)	2/49 (4.08%)	9/30* (30.0%)	**p = 0.001**
Dressing change frequency/week, mean (SD)	4.32 (1.69)	3.54 (1.90)	**p = 0.035**

Legend: No–number; SD–standard deviation; CCAC–community care access centre; TRWHC–Toronto Regional Wound Healing Clinic

*Number of wounds closed refers to the final visit at TRWHC where the denominator representing the total number of patients from the initial cohort has decreased.

Significant improvement in wound closure rates were demonstrated following TRWHC assessment and care (p = 0.001). Once a patient achieved wound closure of their primary wound, they were no longer accounted for in the denominator as they were no longer followed by the TRWHC. Weekly dressing change frequency decreased significantly following TRWHC assessment and follow-up (4.32/week to 3.54/week, p = 0.035). When stratified by CCAC service setting (i.e. in-home, outpatient clinic, or patient self-management), a decreasing trend in weekly dressing change frequency was noted, although it did not achieve statistical significance. Quality of life measures were not assessed on a validated scale at time of CCAC referral and thus could not be directly compared. Notably however, 36.7% (18/49) of the cohort reported improved quality of life scores at their second TRWHC clinic encounter.

As a small proportion of patients had prior history of diabetes education, glycemic control measurements, or footwear assessments at time of CCAC referral (*Table 1)*, these treatment elements were addressed at TRWHC. Diabetes education was planned or provided, including referral to diabetes education programs, and HbA1c tests were ordered for an additional 33% of the cohort at initial assessment. Doppler studies conducted in all patients at the TRWHC demonstrated a triphasic or ‘normal’ waveform in only 22.5% (11/49) of the cohort. Although advanced wound care therapies were requested in a number of patients on referral, implementation of negative wound pressure therapy was recommended in less than 5.0% of the cohort, and only 6.1% of patients were referred for consideration of hyperbaric oxygen therapy after TRWHC assessment (data not shown).

## Discussion

When DFU diagnostic and management measures were assessed between a standard community care model and an interprofessional wound care team model, significant improvements were noted in multiple domains. Specifically, the study demonstrated more precise diagnoses, increased healability classifications, increased vascular and footwear/offloading assessment, enhanced identification of deep and surrounding infection, and improved healing times following interprofessional comprehensive would team assessment. Pain and quality of life scores also trended towards improvement, although they did not reach statistical significance. Wound closure was more readily achieved despite a significantly lower mean follow-up time. Few advanced wound care modalities were deemed required. Our findings are consistent with the results of randomized controlled trials of interprofessional wound teams completed in other jurisdictions that have demonstrated significant improvements in healing rates, pain scores, treatment frequency, and trends to wound closure [25,27].

The TRWHC foot assessments and redistribution results may have been improved further if the primary footwear were dispensed following interprofessional assessment as patients at times had inappropriately dispensed footwear without modifications at time of this assessment. Based on previous data from the Guyana foot care project [24] as well as the primary care reform project in Ontario, integration and coordination of footwear and orthotic dispensing by expert foot care clinicians can improve foot-related outcomes.

Although chronic wounds associated with diabetes mellitus are common, significant variation in diagnostic and management practices exist [28,29]. Notably, before their interprofessional comprehensive assessment a large proportion of patients had no documented history of diabetes education, foot screening, or footwear/offloading device assessment, reflecting a lack of evidence based care in DFU care delivery. The lack of standardization and adherence to best practices may contribute to suboptimal outcomes. Socioeconomic factors have been demonstrated to have a negative impact on diabetes related complications and outcomes [30, 31]. Individuals with diabetes have a 15% lifetime risk of developing a DFUs, with recurrence rates of 70% or higher without effective interventions [9, 32, 33].

Not uniquely in Canada, wound care in the province of Ontario has highly varied referral and care pathways. A survey study to characterize the interprofessional service models for wound care in Ontario identified 49 separate teams across the province’s 14 CCACs. The findings identified the numbers of wound care teams, disciplines of team members, locations, clinical experience, volume and diversity of patients assessed, available resources, and access to key services vastly differed between the teams [29]. A study of the burden of chronic wounds in Ontario estimated that 25% of 22,000 long-stay clients (> 60 days) were persons with diabetes that received care annually through CCAC services [29]. Wound care was provided for a mean duration of 27 months with no noted improvement, and potential worsening, in at least 60% of clients in this time period [34]. Total costs of DFU management based on 2009 estimates are approximately $511,000,000 or $5,678 CAD per DFU with provision of standard community care [17, 19].

This study’s primary strengths were the detailed nature of the review, and the comparison of two contrasting models of DFU management. Weaknesses included the relatively small sample size, limited follow-up time of approximately 3–4 months, and use of two models of wound care which hampered our ability to examine short and long-term outcomes of wound recurrence, and mortality. Although individuals with DFU are prone to rapid changes in clinical status and thus may have had changes in their wound between referral and assessment, this was minimized as they were all seen within ten days of referral. Other limitations included drawbacks inherent to retrospective study designs e.g. vulnerability to information bias (i.e. missing data, misclassification of DFU), selection bias based on the heterogeneity of populations, and confounding factors not accounted for in the data analysis. Although there were significant differences in the components of diabetes wound assessments that were completed in the two models of care (i.e. healing potential, vascular assessment), due to the study design and our limited power, larger prospective studies are needed to further validate the findings.

This is one of the first studies to examine and highlight the differences in care between usually wound care in the community and interprofessional care models for DFU management. The population studied were clients with long-term ulcers not meeting the benchmarks for care and therefore costly to the system. Moving forward, a coordinated approach with evidence based paradigms to diabetic ulcer management at the health system level is critical. Given the geographical size of Ontario, a highly specialized team may be needed in each Local Health Integrated Network. Accessibility can be improved by connecting regional interprofessional wound care teams to expert team hubs via pre-defined and well established referral pathways. (e.g. in Ontario the community health link teams would be connected to the interprofessional team within the local integrated health network). Furthermore, larger prospective studies examining the impact of interprofessional teams on DFU management would assist in optimizing team characteristics including team composition and referral pathways, thereby improving patient outcomes, and decreasing DFU attributed health care costs.

### Conclusion

Our study demonstrates a coordinated community-based interprofessional team approach to DFU management is associated with more accurate and precise diagnoses in comparison to current community based care centres. Successful implementation of DFU management strategies may also benefit from optimizing technological and telehealth strategies for education and provision of care. Moving forward, it will be important for healthcare systems, politicians, policymakers, providers, and payers who inhabit them, to coordinate and integrate care with health care professionals. This integrated care approach with interprofessional teams will improve patient-centred outcomes with enhanced value to the healthcare dollar.
